# Reports of anaphylaxis after coronavirus disease 2019 vaccination, South Korea, 26 February to 30 April 2021

**DOI:** 10.2807/1560-7917.ES.2021.26.33.2100694

**Published:** 2021-08-19

**Authors:** Eunju Lee, Yeon-kyeong Lee, Tae Eun Kim, Insob Hwang, Yeon Haw Jung, Hye Ryeon Lee, Jeongsuk Song, Youngjoon Park, Enhi Cho, Yeon-Kyeng Lee

**Affiliations:** 1 Adverse Event Management Team, Immunization Safety Group, COVID-19 Vaccination Task Force, Korea Disease Control and Prevention Agency, Cheongju-si, South Korea; 2 Adverse Event Investigation Team, Immunization Safety Group, COVID-19 Vaccination Task Force, Korea Disease Control and Prevention Agency, Cheongju-si, South Korea; 3 Immunization Safety Group, COVID-19 Vaccination Task Force, Korea Disease Control and Prevention Agency, Cheongju-si, South Korea

**Keywords:** anaphylaxis, COVID-19 vaccination, Brighton Collaboration case definition, adverse events

## Abstract

The South Korea mass vaccination programme administered 3.8 million doses of COVID-19 vaccinations between 26 February and 30 April 2021. After 173 suspected anaphylaxis reports to the nationwide monitoring system for adverse events following immunisation, 44 anaphylaxis cases were confirmed using Brighton Collaboration case definitions. The rates per million doses were 18.2 cases and 6.2 cases for Vaxzevria and Comirnaty, respectively. Median time of onset was 14 min after vaccination and most cases had recovered at the time of review.

The coronavirus disease (COVID-19) mass vaccination campaign in South Korea started on 26 February 2021, with two available COVID-19 vaccines: Vaxzevria (AstraZeneca, Cambridge, United Kingdom (UK); proprietary name in South Korea: AstraZeneca COVID-19 vaccine) and Comirnaty (BioNTech-Pfizer, Mainz, Germany/New York, United States (US); proprietary name in South Korea: Comirnaty (Tozinameran)). Soon after vaccination, suspected anaphylaxis cases were reported and all reports were assessed using the Brighton Collaboration case definition [1]. We describe the characteristics and outcomes of confirmed anaphylaxis cases following COVID-19 vaccination and compare the two COVID-19 vaccines currently available in South Korea, Vaxzevria and Comirnaty.

## COVID-19 vaccination programme in South Korea

The South Korea Ministry of Food and Drug approved the use of Vaxzevria and Comirnaty vaccines on 10 February 2021 and 5 March 2021, respectively. The first vaccination group, recommended by the Korea Advisory Committee on Immunization Practices (KACIP), included healthcare personnel, vulnerable individuals such as residents at long-term care facilities, and people 75 years and older. The type of vaccine was allocated to each group in accordance with recommended conditions of use while vaccine supply was limited (e.g. Comirnaty vaccines for those 75 years and older with mobility, who were required to visit vaccination centres, Vaxzevria vaccines for residents in long-term care facilities because of on-site vaccination). KACIP changed the age recommendation for Vaxzevria vaccine from people aged 18 years and over to only people aged 30 years and over on 12 April 2021 [2], soon after the European Medicine Agency announced a possible link between the Vaxzevria vaccine and very rare cases of unusual blood clots with low blood platelets [3]. 

Physicians were required to report any adverse events following immunisation (AEFI) to the Immunization Management System (KIMS), the web-based reporting system of the Korea Disease Control and Prevention Agency (KDCA), an integrated system for immunisation registration and passive monitoring of adverse event [4]. Anaphylaxis is a rare, life-threatening adverse reaction that occurs within minutes to hours following a vaccination [5]. Patients receiving the vaccine should be monitored carefully for anaphylaxis. According to the KDCA, individuals developing anaphylaxis after COVID-19 vaccination are contraindicated for a second dose. Brief weekly reports on adverse events following COVID-19 vaccinations have been published at the website (ncv.kdca.go.kr) based on physician reporting. 

## Anaphylaxis following COVID-19 immunisation 

From 26 February to 30 April 2021, a total of 3.6 million COVID-19 vaccine doses were administered in South Korea, including 1,810,255 doses of the Vaxzevria vaccine and 1,779,553 doses of the Comirnaty vaccine, with 173 suspected anaphylaxis cases reported. In total, female recipients had 1.7 times more doses than male recipients (Table 1).

**Table 1:** Vaccination doses and suspected anaphylaxis, South Korea, 16 February–30 April 2021

	Vaxzevria		Comirnaty		Total	
	Vaccinated doses	Suspected anaphylaxis	Vaccinated doses	Suspected anaphylaxis	Vaccinated doses	Suspected anaphylaxis
Total	1,810,255	140	1,779,553	33	3,589,808	173
Age group (years)						
< 30	135,184	31	45,256	4	180,440	35
30–39	266,982	43	40,800	5	307,782	48
40–49	354,837	36	37,092	5	391,929	41
50–59	464,337	20	44,082	3	508,419	23
≥ 60	588,915	10	1,612,323	16	2,201,238	26
Sex						
Female	1,198,118	118	1,082,443	25	2,280,561	143
Male	612,137	22	697,110	8	1,309,247	30

The Korea vaccine injury investigation committee (KVIIC) with an external advisory group, consisting of allergists, immunologists and the KDCA, assessed all reports of allergic reactions and suspected anaphylaxis cases with the Brighton Collaboration case definition criteria and level of certainty algorithm. As part of the routine process for all serious AEFI including life-threatening, death and intensive care-acquired, the epidemiological investigation team in each province investigated the allergic reaction reports and collected dossiers, including medical records, and clinical laboratory records, and interviewed patients or their physicians when suspected anaphylaxis cases reported. The collected dossiers were sent to the KDCA for review by the KVIIC. The KVIIC held a weekly meeting to review AEFI including suspected anaphylaxis cases. 

The Brighton Collaboration case definition for anaphylaxis is divided into the following five categories: Levels 1–3 represent the highest level of diagnostic certainty of a reported anaphylaxis case (with level 1 > level 2 > level 3). Level 4 reports anaphylaxis cases with insufficient evidence to reach diagnostic certainty. Level 5 describes cases that did not meet the case definition. Any suspected cases with only one symptom in the major criteria or with two or fewer mild symptoms without the major criteria in the Brighton Collaboration case definition were classified into level 4. We considered levels 1–3 as anaphylaxis cases with diagnostic certainty. Data analysis was performed using SPSS version 18 (SPSS Inc., Chicago, US).

## Characteristics of confirmed anaphylaxis following COVID-19 vaccination

Of the total 173 acute allergic reactions (anaphylaxis-like reactions) reported to the KIMS, we confirmed 44 anaphylaxis cases (including anaphylactoid reactions). Anaphylaxis cases were reported more frequently in recipients of the Vaxzevria vaccine, with 18.2 confirmed cases per million doses, compared with 6.2 cases per million doses of the Comirnaty vaccine (Table 2). 

**Table 2:** Classification of anaphylaxis and anaphylaxis-like acute allergic reactions in recipients of Vaxzevria (n = 1,810,255 doses) and Comirnaty (n = 1,779,553 doses) COVID-19 vaccines using the Brighton Collaboration case definition, South Korea, 26 February–30 April 2021, South Korea

Brighton Collaboration case definition level^a^	Vaxzevria (n = 140)	Comirnaty (n = 33)	Total (n = 173)
	n	n	n
**1**	**12**	**0**	**12**
**2**	**16**	**9**	**25**
**3**	**5**	**2**	**7**
4	50	10	60
5	57	12	69

^a^The Brighton Collaboration case definition uses combinations of symptoms to define levels of diagnostic certainty. Levels 1–3 represent the highest level of diagnostic certainty that a reported case represents anaphylaxis (with level 1 > levels 2 > level 3); level 4 is a case reported as anaphylaxis with insufficient evidence to meet any of the levels of diagnostic certainty; and level 5 is a case that did not meet the case definition (not a case of anaphylaxis) [1]. This study considered levels 1, 2 and 3 as anaphylaxis cases.

The median age of the patients with anaphylaxis was 37 years (range: 21–84), and 38 of the 44 patients were female. The median time from vaccination to symptom onset was 13 min for the Vaxzevria vaccine (4–1,200 min) and 15 min for the Comirnaty vaccine (5–120 min). With either vaccine, most symptoms occurred within 30 min (33 cases), and none of the cases had symptoms for longer than 24 h. Among the patients with anaphylaxis, 21 patients had one or more histories of allergy to food, drugs or vaccines. Specifically, 20 patients with confirmed anaphylaxis with the Vaxzevria vaccine had an allergic history. The most frequent symptoms reported included nausea/vomiting or a sensation of throat closure. At the time the cases were reviewed, 32 patients had recovered without hospital admission and 12 patients were hospitalised, but no anaphylactic shock or death was reported. Eleven of the 12 hospitalised patients had been discharged at the time of review by the KVIIC. Forty-two patients received one or more treatments such as epinephrine, antihistamine or steroid. Among those receiving injection treatment, intramuscular epinephrine was applied for 37 patients (Table 3).

**Table 3:** Characteristics of reported confirmed cases of anaphylaxis following Vaxzevria (n = 1,810,255 doses) and Comirnaty (n = 1,779,553 doses) COVID-19 vaccination, South Korea, 26 February–30 April 2021

	Vaxzevria (n = 33)	Comirnaty (n = 11)	Total (n = 44)
**Age group in years; median (range)**	**37 (21–66)**	**77 (21–84)**	**37 (21–84)**
< 30	11	3	14
30–39	9	1	10
40–49	7	0	7
50–59	5	0	5
≥ 60	1	7	8
**Sex**			
Female	30	8	38
Male	3	3	6
**Symptom onset; median (range)**	**13 (4–1,200)**	**15 (5–120)**	**14 (4–1,200)**
≤ 15 min	21	6	27
≤ 30 min	25	8	33
> 30 min	8	3	11
**Prior allergic reactions type^a^**			
Drug	8	1	9
Vaccine	2	0	2
Food	13	1	14
**Symptom^a^**			
Sensation of throat closure	18	3	21
Upper airway swelling	16	0	16
Nausea/vomiting	16	5	21
Tachycardia	11	6	17
Difficulty breathing without wheeze or stridor	13	3	16
Angio-oedema	12	3	15
Hypotension	6	5	11
Dry cough	6	1	7
Other	33	18	51
**Received treatments^a^**			
Epinephrine	27	10	37
Antihistamine	22	5	27
Steroid	15	4	19
**Treatment progress**			
Hospitalised	8	4	12
Outpatient/emergency room	25	7	32

^a^ Multiple responses were possible.

## Ethical statement

Given that the current activity was conducted and authorised by the public health authority and the purpose was to disseminate information to the public, and the data are presented in aggregated format, the current study falls under one of the exemption categories by the government regulation for the ethical board review.

## Discussion

The anaphylaxis rates in South Korea following COVID-19 vaccination were not different from those of other countries (Vaxzevria 19.5 per million doses, Comirnaty 13.7 per million doses in the UK [6], Moderna 2.5 cases per million doses and Comirnaty 11.1 cases per million doses in the US [7,8]). However, our data cannot be easily compared with those of previous reports because the reporting systems and vaccination target groups differ. In South Korea, people 75 years or older were given the Comirnaty COVID-19 vaccine, and those accounted for lower reported rates of anaphylaxis. In addition, the Vaxzevria COVID-19 vaccine was not administered to people younger than 30 years because of the risk of thrombosis with thrombocytopenia syndrome [9]. Similar to a previous report from the US [10], anaphylaxis had a higher incidence among women, with 16.7 cases per million doses compared to 4.6 cases per million doses in men. Among 44 confirmed cases, one case occurred following second-dose vaccination in a 30 year-old man. 

A limitation of our study is that the initial vaccination group was composed of healthcare personnel, residents of long-term care facilities and front-line COVID-19 response personnel. Thus, the data obtained from this study are not directly comparable with the general population of South Korea. Furthermore, the data may have included allergic reactions, but all suspected cases of anaphylaxis nationwide were assessed by the KVIIC including allergists and immunologists using the Brighton Collaboration evaluation, based on their medical records. 

Among the suspected anaphylaxis reports, 34.7% (60/173 suspected reports) were assessed as level 4, in most cases because symptoms met only the time criterion after COVID-19 vaccination such as sudden onset and rapid progress without sufficient major/minor criteria for anaphylaxis. Because physicians were required to report life-threatening events including anaphylaxis, a reporting bias may exist. 

The median time of anaphylaxis symptom onset was 14 min after vaccination, and most symptoms occurred within 30 min of vaccination. Eleven had onset after 30 min, nine within 2 h, one within 9 h and the remaining one within 20 h. Most patients received at least one treatment and had fully recovered at the time of review without any deaths. 

## Conclusion 

The KACIP and the KDCA emphasise the previous recommendation [4] that all recipients should be monitored for 15–30 min after vaccination in an observation room at vaccination sites. The observational timeframe should take into account the person’s previous history of allergic reactions. Furthermore, vaccination sites and clinics should ensure preparation of appropriate medical supplies such as epinephrine and have healthcare personnel and observational staff fully aware of anaphylaxis symptoms and the appropriate treatments.
